# Knowledge, Attitudes, and Practices of Medical Professionals Regarding Cervical Cancer Prevention and Human Papillomavirus (HPV) Vaccination: A Cross-Sectional Study

**DOI:** 10.7759/cureus.91754

**Published:** 2025-09-07

**Authors:** Kavyarani C, Athish Kannan Karur, Chaitra C, Prashanth V, Preetha Selvaraj, Samuel Olaniyan, Adewale Oyeneye, Rashmi Priya Murthy, Anwadevi Arun

**Affiliations:** 1 Department of Obstetrics and Gynaecology, Sri Devaraj Urs Academy of Higher Education and Research, Kolar, IND; 2 Department of Obstetrics and Gynaecology, JSS Medical College, Mysore, IND; 3 Department of Obstetrics and Gynaecology, Gadag Institute of Medical Sciences, Gadag, IND; 4 Department of Primary Care, SriSai Pc, Fort Wayne, USA

**Keywords:** cancer cervical, cervical cancer awareness, cervical cancer in india, cervical cancer prevention, family medicine/general practice, healthcare professionals (hcp), hpv vaccine awareness, human papillomavirus (hpv), prevention of cancer, women's health

## Abstract

Introduction

Despite being largely preventable through human papillomavirus (HPV) vaccination and regular screening, cervical cancer (CC) remains a major global public health concern. Healthcare professionals play a pivotal role in advocating for and facilitating these preventive measures. However, literature revealed that the gap in knowledge among the health professionals directly impacts the community. The study evaluated medical professionals' awareness, perceptions, and behaviors related to CC prevention and HPV immunization.

Methodology

An observational cross-sectional study was undertaken at a tertiary care hospital in Kolar, India, between August 2024 and February 2025. After systematic random sampling, 406 healthcare professionals (age >18 years) were selected who satisfied the inclusion criteria and completed a structured questionnaire assessing knowledge, attitudes, and practices (KAP) related to CC and HPV. Data analysis was performed using SPSS software. Categorical variables were expressed as percentages and assessed using the chi-squared test. A p-value of less than 0.05 was deemed statistically significant.

Results

The mean age of the study participants was 26.7±13.1 years. Among them, 65.5% were aged ≤20 years, and 61.6% were female. While 77.5% had heard of CC and 83.2% identified HPV as the cause, knowledge gaps existed regarding screening guidelines and early symptoms. Only 51.2% were aware of pap smears, and 14.2% knew the correct annual screening frequency. Regarding attitudes, 90.1% recognized the medical professional's role in prevention, and 49.2% considered HPV vaccination essential. About 71.4% supported routine HPV immunization, and 66.9% were willing to recommend the vaccine. Around 75.3% identified a lack of awareness as a barrier. Regarding practice, 20.1% routinely performed CC screening, 17.3% were vaccinated against HPV, and 23.6% always recommended HPV vaccination. About 21.1% attended relevant workshops, and 59.1% expressed interest in future prevention events.

Conclusion

This study identifies gaps in knowledge and practice among healthcare professionals regarding CC screening and HPV vaccination, despite generally positive attitudes. Strengthening targeted education and capacity-building is essential to empower them as advocates in reducing the CC burden, particularly in underserved areas.

## Introduction

Cervical cancer (CC) continues to be a major public health concern, ranking among the top three cancers affecting women under 45 years in numerous countries, with nearly 80% of cases reported from low- and middle-income nations [1]. It is the leading cause of cancer-related mortality among women in developing nations [2,3]. Given that the average age at diagnosis is relatively younger compared to other major cancers, CC results in a disproportionately high number of life-years lost [4]. Various screening methods are available to detect CC at its precancerous stage. Notably, CC is unique among malignancies in that it can be prevented through vaccination against the human papillomavirus (HPV), the primary causative agent. Women's knowledge and attitudes play a crucial role in determining their willingness to undergo screening and receive the HPV vaccine, factors that carry significant implications for future generations [5]. As a major global health concern, CC continues to profoundly affect the lives of millions of women worldwide [6].

CC poses a major challenge to public health systems, emphasizing the importance of implementing efficient strategies for prevention, early diagnosis, and treatment. It develops in the cervix, the section of the uterus, and is often preceded by a long-term infection with high-risk types of HPV. CC preventive interventions, such as HPV vaccination and screening, reduce CC-related overall morbidity and mortality. Generally, the prevalence of preventable medical conditions like CC can be significantly reduced by improving the population's knowledge, attitudes, and practices (KAP) [7]. College/university students are especially vulnerable to HPV infections because of their age and sexual habits [8]. By raising public awareness about preventing HPV-induced CC, healthcare professionals can have a big impact. However, as per the available literature, insufficient knowledge poses a threat to this [9]. Medical students, who provide direct patient care, lack sufficient knowledge of CC, HPV, and its vaccine. In addition to advocating for HPV vaccination, students can serve as informational resources for family members, medical professionals, and the general public [10,11]. As per Centers for Disease Control and Prevention (CDC) guidelines, a three-dose HPV vaccine is given to boys and girls at 11-12 years of age in order to prevent HPV infections and their aftereffects. Also, for unvaccinated candidates, catch-up vaccination is advised for males aged 13-21 and females aged 13-26 [12].

Despite the availability of wide screening options, a knowledge gap about CC persists [11,13]. This has led to missing or delayed vaccinations and insufficient knowledge of safe practices, including screening, which results in the delayed diagnosis of CC. Furthermore, cultural beliefs, stigmas, and personal attitudes toward gynecological exams or vaccines remain challenges to overcome in the prevention of CC. The present study was undertaken to evaluate the existing KAP of medical professionals regarding CC screening and the use of HPV vaccination for its prevention.

## Materials and methods

This cross-sectional observational analysis was conducted at RL Jalappa Hospital, affiliated with Sri Devaraj Urs Medical and Nursing College, Kolar, India, which is the largest tertiary referral center for the entire district, serving this rural underserved community for more than three decades. A questionnaire-based survey was performed for a period of six months, from August 2024 to February 2025, among healthcare professionals who were more than 18 years old. Individuals with a current or past diagnosis of gynecological malignancies, with a history of abnormal cervical smear results, or who declined to provide informed consent were excluded from the study.

All participants were approached to voluntarily participate in this study after being provided with a brief overview of its purpose. Systematic random sampling was employed for participant selection. Of the 447 individuals who showed interest in participating, only 406 satisfied the inclusion criteria and were included in the final survey. Information was gathered through a structured, self-administered questionnaire that was formulated after reviewing relevant literature and further refined with expert guidance. The questionnaire (Appendices) was structured to collect pertinent data in line with the study's aims and included seven items assessing knowledge, six evaluating attitudes, and five examining practices concerning CC. All completed forms were reviewed by the principal investigator to ensure accuracy and completeness, and any forms with incomplete or incorrect responses were excluded from the final analysis. The study commenced after obtaining approval from the Central Ethics Committee of Sri Devaraj Urs Academy of Higher Education and Research (approval number: SDUAHER/KLR/R&D/CEC/S/UG/76/2024-25). Participants were verbally briefed about the purpose and procedures of the study in a language they could comprehend. Written informed consent was obtained from all individuals before the commencement of data collection. Participant autonomy and confidentiality were strictly upheld throughout the study.

The sample size was calculated using OpenEpi, an open-access calculator that uses the following formula: $\text{n}=\left( \frac{\text{Z&alpha;}}{2} \right)^{2}\times\frac{\text{p(1&minus;p)}}{\text{d}^{2}}$. Assuming a knowledge prevalence of 84.4% [7] regarding CC, with a 95% confidence interval and a 5% absolute precision, the minimum sample size required for the study was estimated to be 203. Information was gathered using a pre-structured, researcher-designed questionnaire that included sociodemographic variables and evaluated the participants' KAP regarding CC screening and HPV vaccination among healthcare professionals.

Statistical analysis

Data were collected using a case record form, coded, and entered into Microsoft Excel 2016 (Microsoft Corporation, Redmond, Washington, United States). Statistical analysis was performed using IBM SPSS Statistics for Windows, Version 22.0 (IBM Corp., Armonk, New York, United States). Categorical data were presented as counts and percentages. A p-value below 0.05 is considered statistically significant.

## Results

The average age of the study participants was 26.7±13.1 years. Of the total participants, 266 (65.51%) were aged 20 years or younger, followed by 88 participants (21.67%) in the 21-30-year age group and 46 participants (11.34%) in the 31-40-year age group. Only two participants were aged more than 50 years (Table 1). Out of the total, 250 (61.6%) participants were females, and the remaining 78 (34.4%) participants were males.

**Table 1:** Demography of the study participants (n=406)

Variables		Frequency (n)	Percentage (%)
Age (years)	<20	266	65.51
	21-30	88	21.67
	31-40	46	11.34
	41-50	4	0.99
	>50	2	0.49
Educational status	Undergraduate	317	78.1
	Graduate and above	89	21.9
Number of children	None	378	93.1
	One	17	4.2
	More than 1	11	2.7
Department	Medicine	124	30.54
	Allied Health Science (nursing)	282	69.45
Religion	Hindu	227	55.92
	Muslim	61	15.02
	Christian	98	24.13
	Others	9	2.22
	Preferred not to say	11	2.71
Residence status	Lives with family	58	14.3
	Lives in a dormitory	30	7.4
	Lives alone	318	78.3
Family background	Urban	58	14.3
	Rural	348	85.7

Among all participants, 310 (77.4%) were aware of CC, while 338 (83.2%) correctly identified HPV as its primary causative agent. With respect to CC, 232 (57.1%) participants were aware that women aged 20-40 years are most commonly affected. While 264 (65.1%) recognized post-coital bleeding as a symptom, only 22 (5.5%) correctly identified that CC may be asymptomatic in its early stages. Among the participants, 340 (83.7%) were aware that CC is a preventable condition, while 208 (51.2%) knew that the Pap smear is available as a screening tool for its detection. Furthermore, 58 participants (14.2%) were aware that sexually active women should receive Pap smear screening on an annual basis (Table 2).

**Table 2:** Distribution of knowledge regarding cervical cancer among the study participants (n=406)

Variables	Responses	Frequency (n)	Percentages (%)
Heard about cervical cancer	Yes	310	77.4
	No	96	23.6
Causes of cervical cancer	Human papillomavirus	338	83.2
	Genetic factors	26	6.4
	Addiction	24	5.9
	Immunosuppression	14	3.4
	Don't know	4	0.9
Knowledge regarding the most commonly affected age group to cervical cancer	<20 years	36	8.8
	20-40 years	232	57.1
	41-60 years	122	30.1
	>60 years	16	4
Symptoms of cervical cancer	Post-coital bleeding	264	65.1
	Pelvic pain	36	8.8
	Vaginal discharge	60	14.7
	Irregular menstruation	24	5.9
	No symptoms	22	5.5
Cervical cancer is preventable	Yes	340	83.7
	No	66	16.3
Awareness regarding Pap smear as a screening tool	Yes	208	51.2
	No	198	48.8
Knowledge regarding when Pap smear should be done in sexually active women	Annually	58	14.2
	Every 3 years	100	24.6
	Every 5 years	50	12.3
	Don't know	198	48.8

A total of 138 participants (33.9%) believed that CC screening is essential for all women. Furthermore, a large majority, 366 participants (90.1%), expressed the view that the medical professionals should take the lead in CC prevention efforts. Nearly half of the study participants, 200 (49.2%), believed that HPV vaccination was important for the prevention of CC. A total of 290 (71.4%) participants thought that HPV vaccination should be part of the routine immunization schedule. Out of the total, 272 (66.9%) participants were willing to advise the HPV vaccine to patients. A total of 306 (75.3%) participants had the belief that lack of awareness is the barrier for increasing HPV vaccination (Table 3).

**Table 3:** Attitude regarding cervical cancer among the study participants (n=406) HPV: human papillomavirus

Variables	Responses	Frequency (n)	Percentages (%)
Belief that cervical cancer screening is a must for all women	Strongly agree	168	41.3
	Agree	138	33.9
	Neutral	56	13.7
	Disagree	18	4.4
	Strongly disagree	26	6.4
Belief that medical professionals can take the lead for the prevention of cervical cancer	Yes	366	90.1
	No	40	9.9
Belief that HPV vaccination is important for the prevention of cervical cancer	Strongly agree	200	49.2
	Agree	158	38.9
	Neutral	26	6.4
	Disagree	14	3.4
	Strongly disagree	8	1.9
Belief that HPV vaccination should be part of the routine immunization schedule	Yes	290	71.4
	No	116	28.6
Willingness to recommend the HPV vaccine to patients	Yes	272	66.9
	No	134	33.1
Barriers for increasing HPV vaccination	Cost of vaccine	56	13.7
	Fear of side effects	22	5.5
	Cultural or religious beliefs	18	4.4
	Lack of awareness	306	75.3
	Don't know	4	0.9

Among the study participants, a total of 82 (20.1%) candidates have routinely undergone screening for CC in their practice. A total of 70 (17.3%) participants were vaccinated against HPV. Of the total, 96 (23.6%) participants always recommended the HPV vaccine to eligible patients. A total of 86 (21.1%) participants attended workshops/continuing medical education (CME) regarding CC prevention and HPV vaccination. Out of the total, 240 (59.1%) participants were interested in attending a gathering regarding the prevention of CC in the future (Table 4).

**Table 4:** Practice regarding cervical cancer prevention among the study participants (n=406) CME: continuing medical education; HPV: human papillomavirus

Variables	Responses	Frequency (n)	Percentages (%)
Routinely perform screening of cervical cancer in practice	Yes	82	20.1
	No	304	79.9
Vaccinated against HPV	Yes	70	17.3
	No	336	82.7
Recommended the HPV vaccine to eligible patients	Always	96	23.6
	Frequently	112	27.5
	Occasionally	122	30.2
	Never	76	18.7
Attend workshops/CME regarding cervical cancer prevention and HPV vaccination	Yes	86	21.1
	No	320	78.9
Interested to attend function on the prevention of cervical cancer in the future	Yes	240	59.1
	No	166	40.9

## Discussion

The objective of this study was to assess healthcare professionals' KAP concerning strategies for the prevention of CC. Certain strains of HPV are recognized as the primary cause of cervical intraepithelial neoplasia, which can progress to CC [8,14]. HPV types 16 and 18 are the cause of over half of high-grade cervical pre-cancerous lesions and more than three-fourths of CC. The detection and management of premalignant lesions remain the most effective approach to preventing CC, reinforcing its status as a largely preventable disease [8]. Cytological screening, particularly the Pap smear, is one of the most widely accepted methods for the early detection and prevention of CC.

The study highlighted significant gaps in essential knowledge and awareness regarding CC. The average age of participants in the present study was 26.7±13.1 years. Most participants, 266 (65.5%), were aged 20 years or below, with an additional 88 participants (21.6%) falling within the 21-30 age range. Only two individuals in the study population were aged over 50 years. The average age in the present study varied from earlier findings: Zahediet al. [15] and Khanna et al. [16] documented a mean age of 34 years, whereas Kashyap et al. [17] and Easwaran et al. [18] reported mean ages of 19.77±6.708 years and 54.3±9.8 years, respectively. Regarding gender distribution, 250 participants (61.6%) were female, whereas 156 participants (34.4%) were male.

In the current research, regarding knowledge about CC, 310 (77.5%) participants have heard about CC. In total, 338 (83.2%) participants had correct knowledge regarding HPV as the cause of CC. Over half of the participants (57.1%; n=232) were aware that women aged 20-40 years are the most commonly affected age group in CC. Kumari et al. [19] reported that only 6.25% of participants were aware of CC, whereas much higher awareness levels were observed in the studies by Islam et al. [20] and Fitzpatrick et al. [21], with 90.3% and 81.2% of participants, respectively, having heard about the disease. These disparities may be attributed to regional variation, differences in educational levels, and greater cancer awareness within those study populations. In the current study, 264 participants (65.1%) identified post-coital bleeding as a symptom of CC, whereas only 22 participants (5.5%) knew that the disease can present without symptoms in its early stages. The majority of the participants, 340 (83.7%), reported that CC is a preventable disease. A total of 208 participants (51.2%) were aware of Pap smears as a screening tool for CC. Of the total, 58 (14.2%) participants had knowledge that an annual Pap smear was done in sexually active women. Getaneh et al. [22] reported that 20.8% of participants recognized foul-smelling vaginal discharge as a symptom of CC and 20.6% identified irregular vaginal bleeding as a clinical sign.

In our study, a total of 138 (33.9%) participants agreed that CC screening is necessary for all women. Of the total, 366 (90.1%) participants had the belief that the medical professionals can take the lead for the prevention of CC. Nearly half of the participants, 200 (49.2%), believed that HPV vaccination was important for the prevention of CC. A total of 290 (71.4%) participants believed that HPV vaccination should be part of the routine immunization schedule. Among the participants, 272 (66.9%) indicated a willingness to recommend the HPV vaccine to patients. Additionally, 306 (75.3%) identified lack of awareness as a key barrier to HPV vaccine uptake. According to a study by Sakrawal et al. [23], while 86.3% of women did not support universal screening, 63.3% indicated they would undergo screening if given the opportunity. Furthermore, 98.3% of participants were willing to share information about CC and its screening methods. If a Pap smear detected an abnormality, approximately 65% of women stated they would pursue further evaluation, whereas 34% were uncertain about the appropriate next steps.

In this study, among the study participants, a total of 82 (20.1%) routinely perform screening for CC in their practice. In total, 70 (17.3%) participants were vaccinated against HPV, and 96 (23.6%) participants always recommended the HPV vaccine to eligible patients. Only 86 participants (21.1%) had attended workshops or CME sessions focused on CC prevention and HPV vaccination. In this study, 240 participants (59.1%) showed willingness to participate in future educational programs on CC prevention. According to Heena et al. [24], merely 5.6% of individuals, totaling 22 participants, had been vaccinated against HPV. In a similar vein, Chawla et al. [6] reported that 79.44% of participants had not received the CC vaccine. Likewise, Bencherit et al. [25] observed a low acceptance rate among nursing students, with only 26.7% expressing willingness to get vaccinated against HPV.

The National Programme for Prevention and Control of Cancer, Diabetes, Cardiovascular Diseases, and Stroke (NPCDCS), established in 2010, emphasizes cancers that can be managed through primary and secondary prevention strategies [26]. Therefore, this study underscores the importance of educating healthcare professionals about CC screening. Their role is pivotal for creating awareness and educating patients and successfully implementing current and future programs, especially the extension of the NPCDCS into rural communities [26].

The findings of this study provide important perspectives on healthcare professionals' understanding, perceptions, and behaviors related to CC prevention and HPV immunization. The strengths of this study lie in the relevance of its topic, the clarity of its objectives and methodology, the comprehensive presentation of data, and its emphasis on healthcare professionals as key agents in prevention. The inclusion of a larger sample size, particularly with student participants from diverse communities across India, further enhances the validity of the findings.

Nonetheless, certain limitations must be acknowledged. The cross-sectional design restricts the ability to establish causal associations, while the exclusive inclusion of female healthcare professionals from a single tertiary hospital in a rural setting limits the generalizability of the results. In addition, reliance on self-reported data introduces the possibility of social desirability bias, and the absence of a formal evaluation of the questionnaire's validity and reliability further constrains the study's robustness.

## Conclusions

This study reveals notable gaps in KAP among healthcare professionals regarding CC screening and HPV vaccination. Despite recognizing CC as preventable and expressing positive attitudes, awareness of symptoms, guidelines, and vaccination practices was limited, with low uptake of preventive measures. These findings highlight the need for targeted educational and capacity-building initiatives to empower healthcare professionals as key advocates in reducing the CC burden, especially in rural and underserved areas.

## Appendices

Proforma

Section 1: Demographics

1. Age: _______

2. Education status: _______

3. Number of children: _______

4. Department: _______

5. Religion: _______

6. Residence status: _______

7. Family background: _______

Section 2: Knowledge About Cervical Cancer

8. Have you heard about cervical cancer?

- Yes/No

9. What are the main causes of cervical cancer? (Check all that apply)

- Human papillomavirus (HPV)

- Genetic factors

- Addiction (smoking)

- Immunosuppression

- Don't know

10. Which age group is most commonly affected by cervical cancer?

- Below 20 years

- 20-40 years

- 40-60 years

- Above 60 years

11. What are the symptoms of cervical cancer? (Check all that apply)

- Post-coital bleeding

- Irregular menstruation

- Pelvic pain

- Vaginal discharge

- No symptoms in early stages

12. Is cervical cancer preventable?

- Yes/No

13. Are you aware of the Pap smear test as a screening tool for cervical cancer?

- Yes / No

14. How often should Pap smears be done in sexually active women?

- Annually

- Every 3 years

- Every 5 years

- Don't know

Section 3: Attitude Towards Cervical Cancer Prevention and Vaccination

15. Do you believe cervical cancer screening is necessary for all women?

- Strongly agree/Agree/Neutral/Disagree/Strongly disagree

16. Do you think medical professionals should take the lead in advocating for cervical cancer screening and vaccination?

- Yes/No

17. Do you think HPV vaccination is important for preventing cervical cancer?

- Strongly agree/Agree/Neutral/Disagree/Strongly disagree

18. Should HPV vaccination be part of the routine immunization schedule for girls and young women?

- Yes/No

19. Are you willing to recommend HPV vaccination to your patients?

- Yes/No

20. What do you think are the barriers to increasing HPV vaccination rates? (Check all that apply)

- Cost of the vaccine

- Fear of side effects

- Cultural or religious beliefs

- Lack of awareness

- Lack of access to healthcare

- Other (Please specify)

Section 4: Practice Regarding Cervical Cancer Prevention

19. Do you routinely perform cervical cancer screening in your practice?

- Yes/No

20. Have you been vaccinated against HPV?

- Yes/No

21. How often do you recommend HPV vaccination to eligible patients?

- Always

- Frequently

- Occasionally

- Never

22. Have you attended any workshops or CME programs on cervical cancer prevention and HPV vaccination?

- Yes/No

23. Would you be interested in attending a future program on cervical cancer prevention and HPV vaccination?

- Yes/No
